# Ensemble bias correction of climate simulations: preserving internal variability

**DOI:** 10.1038/s41598-021-82715-1

**Published:** 2021-02-04

**Authors:** Pradeebane Vaittinada Ayar, Mathieu Vrac, Alain Mailhot

**Affiliations:** 1 Institut national de la recherche scientifique, Centre Eau Terre Environnement, Quebec, G1K 9A9 Canada; 2 Laboratoire des Sciences du Climat et l’Environnement (LSCE-IPSL) CNRS/CEA/UVSQ, UMR8212, Université Paris-Saclay, 91190 Gif-sur-Yvette, France

**Keywords:** Atmospheric science, Climate change, Statistics, Scientific data

## Abstract

Climate simulations often need to be adjusted (i.e., corrected) before any climate change impacts studies. However usual bias correction approaches do not differentiate the bias from the different uncertainties of the climate simulations: scenario uncertainty, model uncertainty and internal variability. In particular, in the case of a multi-run ensemble of simulations (i.e., multiple runs of one model), correcting, as usual, each member separately, would mix up the model biases with its internal variability. In this study, two ensemble bias correction approaches preserving the internal variability of the initial ensemble are proposed. These “Ensemble bias correction” (EnsBC) approaches are assessed and compared to the approach where each ensemble member is corrected separately, using precipitation and temperature series at two locations in North America from a multi-member regional climate ensemble. The preservation of the internal variability is assessed in terms of monthly mean and hourly quantiles. Besides, the preservation of the internal variability in a changing climate is evaluated. Results show that, contrary to the usual approach, the proposed ensemble bias correction approaches adequately preserve the internal variability even in changing climate. Moreover, the climate change signal given by the original ensemble is also conserved by both approaches.

## Introduction

Bias correction of climate model simulations is often needed prior to impact studies^1^. Indeed, impact models (e.g., in hydrology, ecology, agriculture) are usually calibrated with observations. The models usually present discrepancies (or biases) from these observations and using raw climate models outputs in impact models may result in meaningless results (e.g., for hydrological model^2^). Biases in climate models are often characterised by differences in statistical distributions between observed and simulated series. Many statistical bias correction (BC) methods have been developed to correct biases in simulations and get simulated series with appropriate statistical properties. Currently, a large majority of BC methods aims at adjusting the mean, the variance and quantiles of a given climate variable distribution. Multiple quantile-based BC methods are actually available^3–7^.

However, when using a single climate model simulation to calibrate a BC method, what is assimilated to a bias may actually be uncertainties. This should thus be treated in a different way. There are various sources of uncertainty in climate models: emission scenario uncertainty, model uncertainty, and climate internal variability^8^. Emission scenario (also called forcing scenario) uncertainty is related to the inability to foresee the greenhouse gas emissions in the future. It is usually evaluated with climate projections generated for different representative concentration pathway (RCP) obtained from various socioeconomic projections^9^. Model uncertainty is related to the various responses resulting from the different model representations of the Earth climate system^10^. For a given forcing scenario, model uncertainties can combine general circulation models (GCMs) and other models such as downscaling models (Regional Climate Models, RCMs and/or statistical downscaling methods, e.g., Vaittinada Ayar et al.^11^) as well as impact models (e.g., hydrological models) uncertainties.

Model uncertainty can be estimated using different modelling chains, combining for instance different climate, downscaling, and impact models (IPCC^12^). The internal variability represents the natural variability of the climate due to its chaotic and nonlinear nature and can be present even in transient climate^13,14^. Whereas forcing scenario and model uncertainties should be reduced with adjustments to future emissions and improvements our knowledge in the representations of the earth system models, climate internal variability is related to the chaotic nature of the climate system and remains irreducible^14,15^. Many studies have estimated these different uncertainties using simulation ensembles, run with different forcing scenarios, various GCM or RCM models and more rarely with multiple initial and/or boundary conditions^8,16,17^.

When applying BC methods to adjust the simulations, the different sources of uncertainties contributing to the biases are not differentiated. Thus, the question is: should all the bias components should be treated indiscriminately? More precisely, should bias due to model uncertainties and bias related to internal variability (the latter being hereafter referred to as IV) be treated at the same time? For instance, the IV of an RCM can be obtained by a multi-member simulation ensemble of that RCM i.e., which consist of a set of RCM simulations using different initial or boundary conditions called members. Such an ensemble can be very insightful to perform sensitivity analyses on a hydrological model or generate an ensemble of hydrological simulations^18^. However such ensemble needs to be bias-corrected before it can be used for hydrological modelling. However, one caveat of actually available quantile-based BC approaches is that they are not able to discriminate the respective contributions to total bias originating from model uncertainties and those associated to internal variability. Therefore, correcting each member separately, i.e., without taking into consideration IV, would reduce (or at least change) the IV of the bias-corrected ensemble, which could be inappropriate for impact studies where the impact of IV needs to be assessed.

The aim of this study is to develop a BC method that preserves the IV of the ensemble. The proposed method is based on the “Cumulative Distribution Function-transform” (CDF-t) initially developed to downscale wind^19^, and later applied to correct and/or downscale univariate simulated distribution. Many applications of the approach have been made since^20–23^, and variant versions also developed^24,25^. Inspired from CDF-t, two ensemble BC methods are implemented and compared at two sites, Chicago and New York where observed and multi-member RCM simulated precipitation and temperature series are available.

Section “Model and reference data” presents the observational and simulated datasets used in this study. The methodology is presented in section “Ensemble bias correction: EnsBC”. The results are shown in section “Results” and summarised and discussed in section “Conclusions and discussions”.

## Model and reference data

The “Climate change and hydrological Extremes” (ClimEx) project has produced a large ensemble (LE) of 50 high-resolution RCM simulations over two domains, North-eastern North America and Europe, at $0.11^{\circ } \times 0.11^{\circ }$ spatial resolution ($\sim$12 km; for details see Leduc et al.^26^).

Boundary conditions used to drive those high-resolution RCM simulations were provided by the Canadian Fourth generation Atmospheric Global Climate Model (CanESM2^27,28^). The 50 independent CanESM2-LE runs^29,30^ were simulated under the Representative Concentration Pathway 8.5 (RCP8.5) emission scenario^9^. Random perturbations of the initial state of cloud-overlap parameters were applied to generate the 50 ensemble members with all the other settings (e.g., forcing scenario and model parameters) unchanged^30^. This 50-member ensemble has been generated over the period 1950–2099 by the 5th generation Canadian Regional Climate Model (CRCM5^31,32^) and is hereafter referred to as the CRCM5 Large-Ensemble (CRCM5-LE). A four-year spin up period was discarded from each CRCM5-LE member resulting in 146-year series (1954–2099) at each of the $280 \times 280$ grid points of North-America. Specific details about CRCM5 dynamics, the sub-grid model parametrisation and the ClimEx experiment set-up and validation have been thoroughly described^26,33^. Various studies using the CRCM5-LE have been carried out in historical and future climate^26,34–36^.

Records from weather stations part of the HadISD^37,38^ database (HadISD data product version 3.1.1.202004p) located in the simulation domain are used as reference datasets. Hourly series are selected according to data availability over the period 1961–1990. Only stations with at least 20 years with less than 5% annual missing values are considered. Two stations are finally selected, one located at Chicago O’Hare airport and the second one at New York LaGuardia airport.

For each station, the precipitation and temperature time series of the CRCM5 grid-cell containing the stations are extracted. In the following, the 1961–1990 period will be used as the calibration period.

## Ensemble bias correction: EnsBC

The proposed methods are based on the “Cumulative Distribution Function-transform” (CDF-t) bias correction (BC) method^19^. The CDF-t method consists in relating the CDF of the simulated (CRCM5-LE) variable to the CDF of the reference (station) variable (i.e., here precipitation or temperature recorded series at Chicago or New York stations). CDF-t is a quantile–quantile method that takes into account the potential change in the modelled CDF from the calibration (i.e. historical) period to the projected time period. This is done through the definition of a transfer function *T*, linking the simulated and reference CDFs.

Let $F_{S_C}(x)$ and $F_{M_C}(x)$ define respectively the CDFs of variable *x* (e.g. temperature or precipitation) at one station (here Chicago or New-York) for the observed and simulated series over the calibration period, and $F_{S_P}(x)$ end $F_{M_P}(x)$ the corresponding CDFs over the projection period.

The transfer function *T* is defined by:

1 $$\begin{aligned} T\left( F_{M_C}(x)\right) =F_{S_C}(x) \Leftrightarrow T(u)=F_{S_C}\left( F^{-1}_{M_C}\left( u\right) \right) \end{aligned}$$

with *u* in [0, 1]. As *T* is assumed to be valid even in a climate different from the calibration one, it can be applied to link the simulated and reference CDFs in the projection period:

2 $$\begin{aligned} T(F_{M_P}(x)) = F_{S_P}(x). \end{aligned}$$

Combining (1) and (2) allows to estimate the CDF for the bias corrected variable *x* for each individual member *m* (BCIND) over the projection period, ${\widehat{F}}_{S^{(m)}_P}(x)$, using the expression:

3 $$\begin{aligned} (\text {BCIND})\quad {\widehat{F}}_{S^{(m)}_P}(x) = F_{S_C}\left( F^{-1}_{M^{(m)}_C}\left( F_{M^{(m)}_P}(x)\right) \right) . \end{aligned}$$

Based on this explicit formulation of ${\widehat{F}}_{S^{(m)}_P}$, a quantile mapping between ${\widehat{F}}_{S^{(m)}_P}(x)$ and $F_{M^{(m)}_P}(x)$ can then be performed to derive the bias-corrected data over the projection period. The modelled bias-corrected quantile, ${\widehat{x}}_{S^{(m)}_P}$, is obtained by applying the inverse CDF estimated in Eq. (3):

4 $$\begin{aligned} {\widehat{x}}_{S^{(m)}_P}={\widehat{F}}^{-1}_{S^{(m)}_P}\left( F_{M^{(m)}_P} (x_{M^{(m)}_{P}})\right) . \end{aligned}$$

While the traditional quantile-mapping approach will use the formulation ${\widehat{x}}_{S^{(m)}_P}=F^{-1}_{S_C}\left( F_{M^{(m)}_C}(x_{M^{(m)}_{P}})\right)$ (i.e., based on two distributions characterising the calibration period), the CDF-t method relies on two distribution distributions characterising the projection period (Eq. (4)). Thus, CDF-t takes into account the potential evolutions (or non-stationarities) of the model distribution between the calibration and projection periods. This property stands for all the ensemble BC methods further presented. Additional details can be found in Vrac et al.^5^.

In the following, this formulation of the CDF-t method is used to bias-correct temperature. A slightly different version is applied for precipitation. A “threshold adaptation” (TA) technique is first applied to correct the frequency of rain occurrence followed by the application of the CDF-t method to the strictly positive precipitation intensities^25^.

This method is applied to each member *m* of the CRCM5-LE and is hereafter referred to as BCIND. Note that BCIND is the classical way to correct biases of ensembles of RCM simulations and is considered as the reference to which two proposed methods of ensemble bias correction (EnsBC) are compared.

In a first version, in order to preserve the IV in the BC results, the CDF of the variable of interest over the reference period and for each member *m* of the CRCM5-LE, $F_{M^{(m)}_C}$ in Eq. (1), is replaced by the CDF of the entire ensemble over the reference period $F_{M-Ens_C}$. This approach is labelled as BCENS hereafter and Eq. (3) thus becomes:

5 $$\begin{aligned} (\text {BCENS})\quad {\widetilde{F}}_{S^{(m)}_P}(x) = F_{S_C}\left( F^{-1}_{M-Ens_C}\left( F_{M^{(m)}_P}(x)\right) \right) . \end{aligned}$$

where ${\widetilde{F}}_{S^{(m)}_P}(x)$ is the CDF for the bias corrected variable *x* for each member *m* taking into account the variability among the various members by using the ensemble distribution.

The second version is a slight modification of BCIND in which, for each member *m* of the CRCM5-LE, the modelled bias-corrected quantile, ${\widehat{x}}_{S^{(m)}_P}$ obtained in Eq. (4), is modified in order to account for the IV over the historical period. Assuming a stationary climate over the 30-year reference period, a difference $\Delta$ is explicitly added to ${\widehat{x}}_{S^{(m)}_P}$. For the given probability $F_{M^{(m)}_P}(x_{M^{(m)}_{P}})$ over the projection period, this difference $\Delta$ corresponds to the absolute difference between the quantile of the member *m* and that of the entire ensemble quantile over the calibration period:

$$\begin{aligned} \Delta = {F}^{-1}_{M^{(m)}_C}\left( F_{M^{(m)}_P}(x_{M^{(m)}_{P}})\right) - {F}^{-1}_{M-Ens_C}\left( F_{M^{(m)}_P}(x_{M^{(m)}_{P}})\right) . \end{aligned}$$

$\Delta$ allows to “reintroduce” the IV of the original CRCM5-LE lost (or changed) with BCIND for each quantile. Hence, the following expression, for the approach hereafter referred to as BCIV, is derived for each member *m* of the CRCM5-LE:

6 $$\begin{aligned} (\text {BCIV}) \left\{ \begin{array}{ll} {\widetilde{x}}_{S^{(m)}_P} &= {\widehat{x}}_{S^{(m)}_P} + \Delta \\ \Leftrightarrow &={\widehat{F}}^{-1}_{S^{(m)}_P}\left( F_{M^{(m)}_P}(x_{M^{(m)}_{P}})\right) + {F}^{-1}_{M^{(m)}_C}\left( F_{M^{(m)}_P}(x_{M^{(m)}_{P}})\right) - {F}^{-1}_{M-Ens_C}\left( F_{M^{(m)}_P}(x_{M^{(m)}_{P}})\right) \end{array} \right. \end{aligned}$$

Note that for precipitation, the “occurrence-intensity” separation is also performed via the threshold adaptation technique for both BCENS and BCIV approaches. In addition, Eq. (6) is valid for intensive variables such as temperature. For extensive variables such as precipitation, the addition/subtraction operators in the previous equation must be replaced by multiplication/division operators to define a function preserving the ensemble variability in quantiles.

## Results

The three bias correction approaches, BCIND, BCENS, and BCIV, are applied at both stations, Chicago and New York, for both variables, temperature and precipitation, on hourly series but on a month-by-month basis. Five projection periods are considered: 1961–1990 (reference period), 1991–2019, 2020–2039, 2040–2069, and 2070–2099. However, for the sake of brevity, the results are mostly focused on the calibration (1961–1990) and the 2040–2069 periods.

### Monthly mean and internal variability

**Figure 1:**
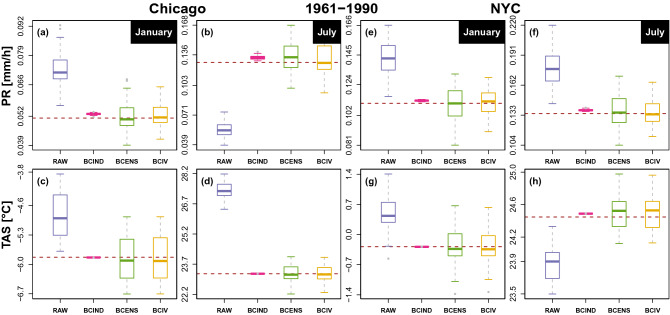
January (**a**,**c**,**e**,**g**) and July (**b**,**d**,**f**,**h**) monthly mean of hourly precipitation (top) and temperature (bottom) for Chicago (**a**–**d**) and New York (**e**–**h**) over the 1961–1990 period for the original (blue) and bias-corrected 50-member CRCM5-LE (purple: BCIND, green: BCENS and yellow: BCIV). Dashed lines represent the observed mean.

The performance of the different EnsBC methods are first assessed through the hourly monthly mean. Figure 1 shows the boxplots of the 50-member ensemble monthly mean hourly temperature and precipitation in January and July at Chicago and New York.

The size of the boxplots measures the internal variability for raw and bias-corrected CRCM5-LE while the medians correspond to the bias of the CRCM5-LE and bias corrected ensembles. As expected, IV is almost completely eliminated (no dispersion of bias corrected values) by the classical BCIND method. These results advocate for the use of EnsBC methods. The values for all months are given in Fig. S1 to S4 of the Supplementary material for the calibration and 2040–2069 periods along with the monthly probability and mean of hours with precipitation ($> 0$ mm).

**Figure 2:**
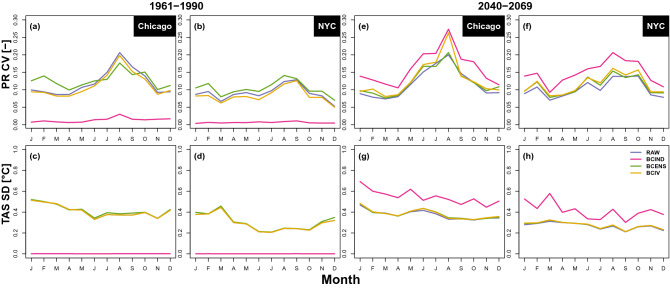
Monthly CVs of hourly mean precipitation (top) and temperature SD (bottom) for Chicago (**a**,**c**,**e**,**g**) and New York (**b**,**d**,**f**,**h**) over the 1961–1990 (**a**–**d**) and 2040–2069 (**e**–**h**) periods. The blue, purple, green and yellow lines respectively represent the CV (for precipitation) and SD (for temperature) of the original (RAW) and the BCIND, BCENS and BCIV corrected ensembles.

In order to thoroughly compare the IV of each ensemble, the coefficient of variability (CV) of the monthly mean precipitation, defined as the standard deviation divided by the median of the ensemble) and standard deviation (SD) of the temperature are computed. Figure 2 shows the monthly precipitation CV and temperature SD for the raw CRCM5-LE and bias-corrected ensembles over the 1961–1990 and 2040–2069 periods for Chicago and New York.

Whether for precipitation or for temperature, BCIND almost completely wipes out the IV of the CRCM5-LE. This was expected since all members are bias-corrected separately using a common reference dataset. However, surprisingly, IV for the 2040–2069 period after applying BCIND method is larger than the raw ensemble for both temperature and precipitation. This can be related to overfitting issues. Indeed, in BCIND, each member is corrected separately which only accounts for the variability of only one member. Thus, a part of the variability is blended to the signal of change in future projections. Hence, the BCIND corrections mixed the climate change signal and the internal variability, resulting in a larger IV in future climate. On the contrary, BCENS and BCIV preserve the IV efficiently, BCIV slightly outperforming BCENS for precipitation in reference period, while it is the opposite for the future period. For temperature, both methods give very good results as projected IV is similar to the projected IV of the original CRCM5-LE.

For precipitation, the CV are in general slightly overestimated (except for BCIV over the calibration period) but remain close to the CV of the CRCM5-LE. These differences may be explained by the large discrepancies between CRCM5-LE and bias-corrected probabilities of occurrence of hourly precipitation (cf. Fig. S1–S4 of the supplementary material). In August for the future period there is a drop of performance in Chicago for BCIV while there is almost no bias in rainfall occurrence (cf. Fig. S1). BCIV is a variant of BCIND and therefore it “inherits” its larger IV over projection period. For precipitation the ratio,

$$\begin{aligned} \Delta =\frac{{F}^{-1}_{M^{(m)}_C}\left( F_{M^{(m)}_P}(x_{M^{(m)}_{P}}) \right) }{{F}^{-1}_{M-Ens_C}\left( F_{M^{(m)}_P}(x_{M^{(m)}_{P}})\right) }, \end{aligned}$$

is supposed to correct this feature. However, August precipitation intensities in Chicago present the largest biases and very small amount and spread for the raw ensemble. This makes $\Delta$ really close to 1 and unable to correct the over-dispersion of BCIND.

### Hourly quantiles and internal variability

**Figure 3:**
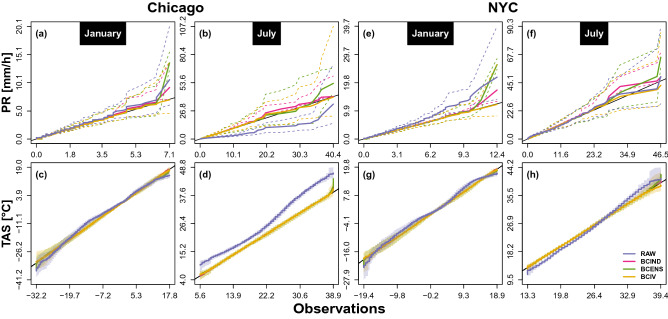
Hourly precipitation amount (top) and temperature (bottom) quantile–quantile plots at Chicago (**a**–**d**) and New York (**e**–**h**) over the 1961–1990 periods for January (**a**,**c**,**e**,**g**) and July (**b**,**d**,**f**,**h**). The blue, purple, green and yellow solid lines represent respectively the original and the BCIND, BCENS and BCIV corrected ensembles median of the simulated quantiles associated to the same probability. For each ensemble, the spread of the simulated quantiles associated to same probability is represented by dashed lines for precipitation and colour shades for temperature.

The capability of the EnsBC methods to reproduce the original IV is then assessed at a quantile level. Figure 3 presents quantile–quantile plots the January and July hourly precipitation and temperature over the calibration period.

The results show that the precipitation quantile spread of the ensemble simulated precipitation quantiles associated to a same probability are located below and above contains the first bisector, except for the BCIND corrected ensemble, for which the bisector is below the spread. For temperature, the bias-corrected ensemble matches the first bisector but it is hard to visually asses the variability of the simulated quantiles since the quantile spreads are much smaller.

**Figure 4:**
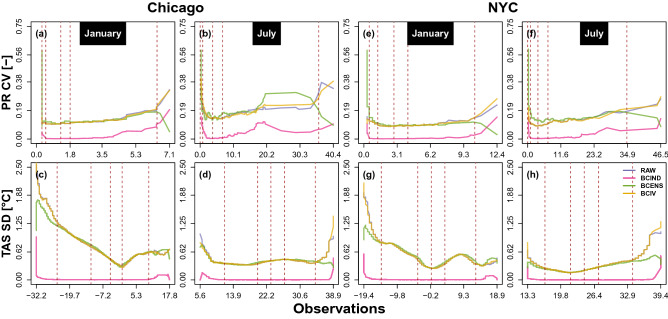
Precipitation CV (top) and temperature SD (bottom) of the simulated hourly quantile associated to the same probability over the 1961–1990 period of in function of the observed quantiles associated to the same probability at Chicago (**a**–**d**) and New York (**e**–**h**) for January (**a**,**c**,**e**,**g**) and July (**b**,**d**,**f**,**h**). The blue, purple, green and yellow solid lines represent respectively the raw and the BCIND, BCENS and BCIV corrected ensembles. Vertical dashed lines represent the observed 95th, 97.5th, 99th, 99.5th and 99.99th precipitation percentiles and the first, 25th, 50th, 75th and 99th temperature percentiles).

To asses more precisely the quantile IV, the CV (resp. SD) of the simulated precipitation (resp. temperature) quantile associated to each given probability are computed and represented against the corresponding observed quantiles in Fig. 4.

As expected from the analysis of the quantile–quantile plots, BCIND clearly fails to reproduce the ensemble quantile variability for both stations and both variables. Losing this variability removes the most important added-value of a multi-member ensemble, even though the bias is corrected. BCENS and BCIV are better performing to preserve the quantile IV for precipitation and temperature. BCIV has a clear advantage for the extreme quantile, i.e., both low- and high-probability CVs for precipitation and SDs for temperature are much closer to raw ensemble corresponding CV and SD than the BCENS corrected ensemble. Figure S5 of the supplementary material gives the equivalent to Fig. 4 but for monthly quantiles. The conclusions are the same as for the hourly quantiles.

### Climate change signal and internal variability

As previously mentioned, the individual CRCM5-LE ensemble members are constrained by boundary conditions with identical radiative forcing and parametrisation. Only the initial state of cloud-overlap parameters are different. After the spin-up period, each member evolves chaotically and the spread is resulting from the IV alone. The total trends stemming from the individual model realisations can be partitioned into contributions from the response to the external forcing and the IV. In order to illustrate if the EnsBC methods have actually preserved the contribution of the IV to climate change signal, the signal-to-noise ratios (SNR) have been calculated. SNR is defined as the ratio between the mean and the standard deviation of the trends from each member of the ensemble^39^:

$$\begin{aligned} \text {SNR}=\frac{\text {mean(trends)}}{\text {sd(trends)}}. \end{aligned}$$

**Figure 5:**
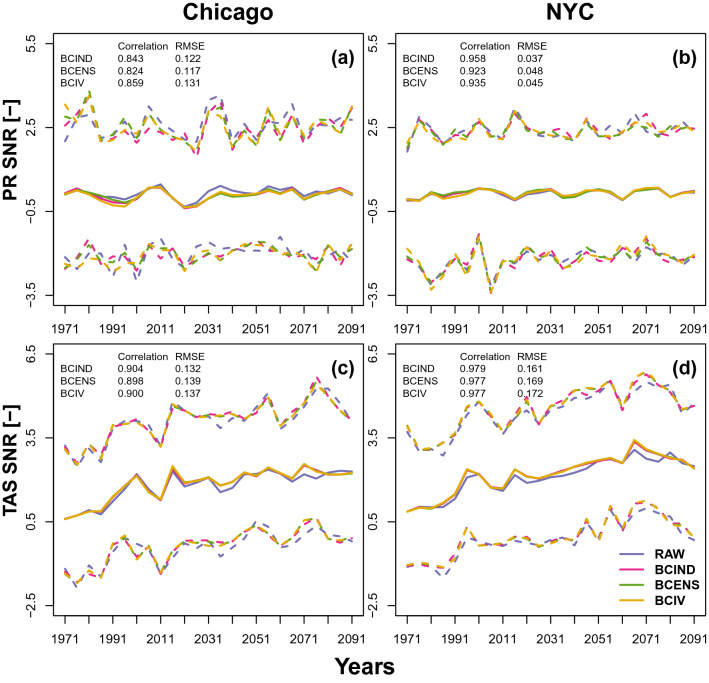
SNR for annual total precipitation (**a**,**b**) and mean temperature (**c**,**d**) over the 1961–2099 period for Chicago (**a**,**c**) and New York (**b**,**d**). The blue, purple, green and yellow solid lines represent respectively the raw and the BCIND, BCENS and BCIV corrected ensembles SNR for each 20-year periods. The dashed lines represent the maximum and minimum SNR boundaries defined respectively as $\frac{\text {max(trends)}}{\text {sd(trends)}}$ and $\frac{\text {min(trends)}}{\text {sd(trends)}}$.

The trends are estimated using the non-parametric Theil–Sen estimator^40^ which considers the median of the slopes over all pairs of sample points as a good estimator of the slope of a linear trend over a given period. Figure 5 represents the SNR estimated for each 20-year sliding window (shifted every five years) over the 1961–2099 period for annual total precipitation and mean annual temperature for the raw and bias-corrected ensembles. The SNR variations of the raw ensemble are well preserved by all the bias corrected ensembles for both variables and both stations. The dashed lines representing the maximum and minimum SNR boundaries defined respectively as $\frac{\text {max(trends)}}{\text {sd(trends)}}$ and $\frac{\text {min(trends)}}{\text {sd(trends)}}$ also show a good preservation of the raw ensemble SNR boundary variations by all the bias corrected ensembles. In order to asses the reproduction of the SNR for extremes, the same exercise has been conducted for annual maximum precipitation and temperature instead of annual total precipitation and mean temperature. The same as Figure 5 for the maxima are given Figure S6 of the supplementary material. The conclusions for the maxima are the same. This means that the three tested BC approaches preserve the IV contribution to the trend of the raw simulations. If this was expected for BCIND with the CDF-t method^22^, this result also indicates that accounting for the IV in the BCENS and BCIV EnsBC approaches still allows the corrections to preserve the main climate change signal and the trends.

## Conclusions and discussions

The aim of this study is to develop ensemble bias correction (EnsBC) methods that preserve the internal variability of a multi-member ensemble after the corrections. Such BC methods are interesting in the purpose of using these simulations to feed impacts models whilst benefiting from the added-value of an multi-member ensemble. To this end, three strategies of EnsBC – both relying on an arbitrarily chosen BC method (here, CDF-t) – are implemented: (i) the BC is performed for each member individually (BCIND), which is the classical way to perform an EnsBC; (ii) the BC is performed for each member using the CDF of the entire ensemble as calibration model CDF (BCENS); and (iii) the BC is performed for each member, explicitly accounting for the internal variability (BCIV). An application of the different EnsBC strategies is performed to correct precipitation and temperature time series simulated from the CRCM5-LE ensemble, with respect to two weather station time series on station data at Chicago and New York. Their ability to reproduce the IV is assessed regarding the variability of the monthly mean and that of quantiles. The preservation of the trends after the corrections is also evaluated.

Results show that the BCIND approach completely fails in the preservation of the ensemble internal variability, both in terms of monthly mean and quantiles, while BCENS and BCIV are successful to provide bias corrections respecting the ensemble IV. BCIV is only slightly better than BCENS but has a clear advantage regarding the preservation of the variability for extreme quantiles. The three EnsBC strategies preserve the IV contribution to climate change signal of the raw ensemble. Based on these conclusions, BCIV seems to be the most suited approach to perform an EnsBC.

However, the main results of this study could be further investigated in various ways. First, the EnsBC strategies proposed in this paper are only based on one univariate BC approach, CDF-t. The same methodology has to be tested for other univariate but also for multi-variate BC methods to better understand the contribution of the specific BC method to the robustness our our findings. Multivariate BC corrects the inter-variable, spatial and/or temporal correlation in addition to the distributional properties (cf. François et al.^7^, and the reference therein for more insights on BC).

Moreover, the methodology developed in this study is only applied to one multi-member ensemble to preserve its IV. The question is still open for the inter-model variability in the case of a multi-model ensemble. For discussion purposes, let us consider that there are many multi-member ensembles. EnsBC can be applied either (i) to all the simulations of all the ensemble together, or (ii) to each ensemble separately. In the first case, the gap between each ensemble would be maintained, which means that part of the biases due to the model uncertainties would remain untouched. In the second case (what is done in this study), the median of each ensemble matches the observations (over the calibration period), which implicitly means that biases are fully comprised in the model uncertainties. Both cases are not necessarily desirable. A way to evaluate what is actually corrected by BC methods and what has to be corrected is needed.

Also, the evaluation of the EnsBC results could be done through ensemble variability partitioning and estimation using, for instance, “Analysis of variance” (ANOVA^17^). Such analyses before and after BC would help to quantify the contribution of the different uncertainties and which ones of them are reduced after EnsBC.

Last but not least, since RCMs simulations are eventually meant to be used as inputs in impact models, added-value and/or any undesired effect of EnsBC methods can be assessed, for instance through hydrological models. Combining a variability analysis and impacts modelling should give insights on what needs to be corrected or not.

## Supplementary information

Supplementary Material 1
