# Supplementary material for: Ensemble bias correction of climate simulations: preserving internal variability
Source: Sci Rep. 2021 Feb 4;11:3098. doi: 10.1038/s41598-021-82715-1 (PMC7862270; doi:10.1038/s41598-021-82715-1)
Supplement: Supplementary file 1 — Supplementary Material 1 [file 41598_2021_82715_MOESM1_ESM.pdf]

Ensemble bias correction of climate simulations: preserving internal variability. Supplementary material

Pradeebane Vaittinada Ayar[1,*], Mathieu Vrac[2], and Alain Mailhot[1]

[1]Institut national de la recherche scientifique, Centre Eau Terre Environnement, Québec, G1K 9A9 Canada
[2]Laboratoire des Sciences du Climat et l'Environnement (LSCE-IPSL) CNRS/CEA/UVSQ, UMR8212, Université Paris-Saclay, Gif-sur-Yvette, 91190 France
[*]pradeebane@laposte.net

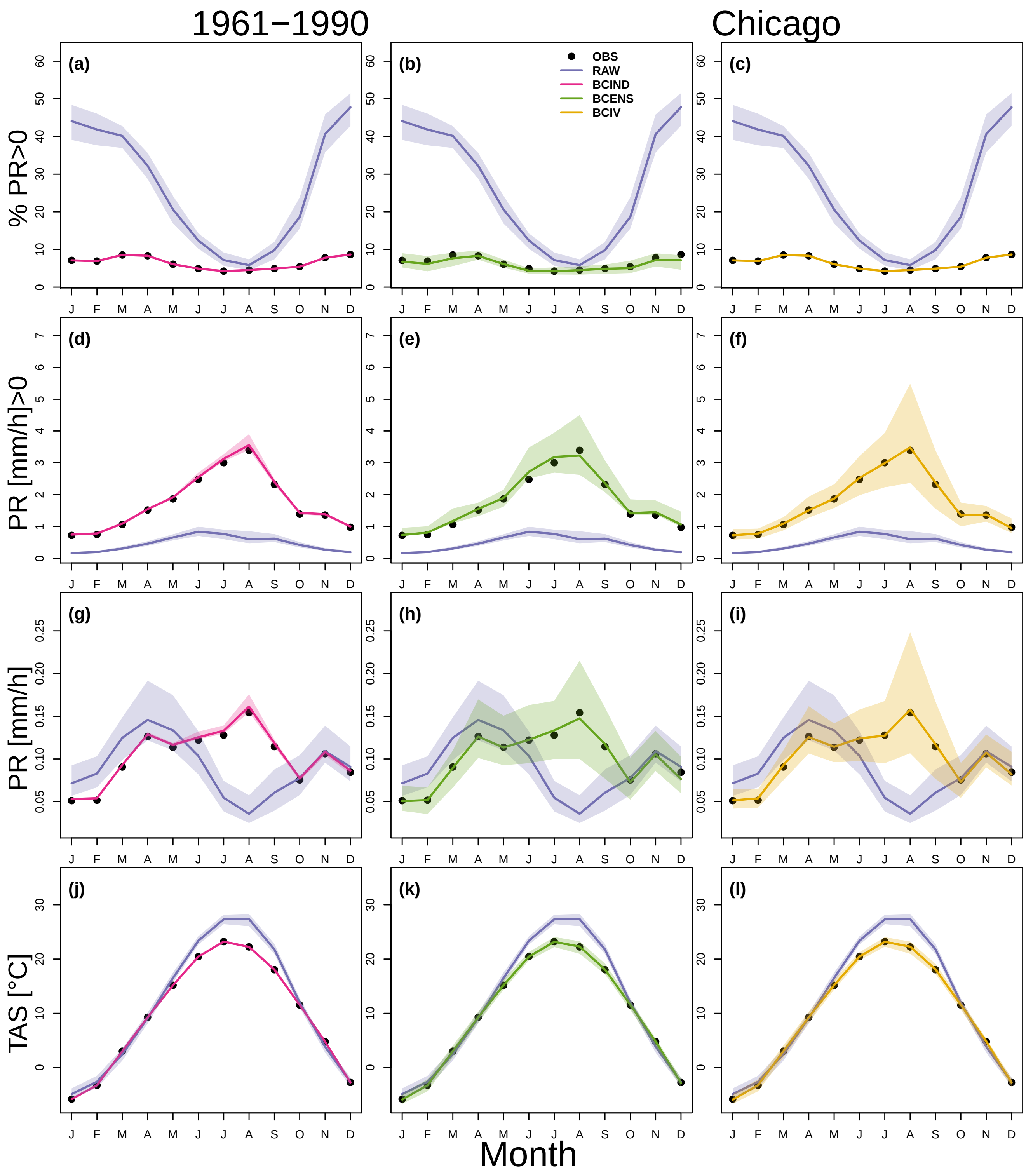

Figure S1. Monthly hourly rainfall probability ((a)-(c)), monthly mean of rainy hours ((d)-(f)), monthly mean rainfall amount ((g)-(i)) and monthly mean temperature ((j)-(l)) at Chicago over the 1961-1990 period of the ensemble corrected with BCIND ((a),(d),(g),(j)), BCENS ((b),(e),(h),(k)) and BCIV ((c),(f),(i),(l)). Observations statistics are represented by black dots, blue represents the raw CRCM5-LE ensemble. The solid lines represent the median of the ensemble.

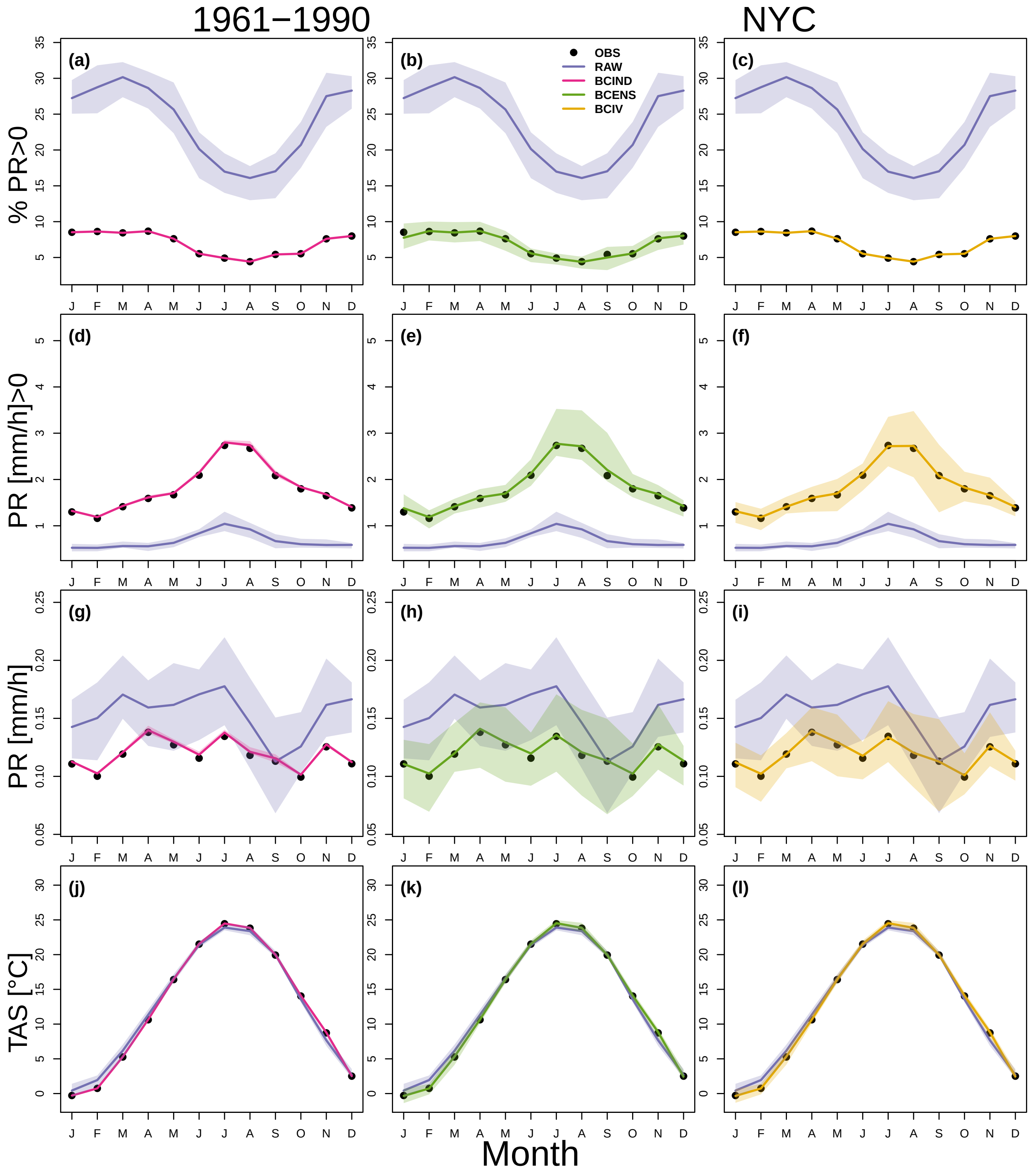

Figure S2. Idem Figure S1 at New York.

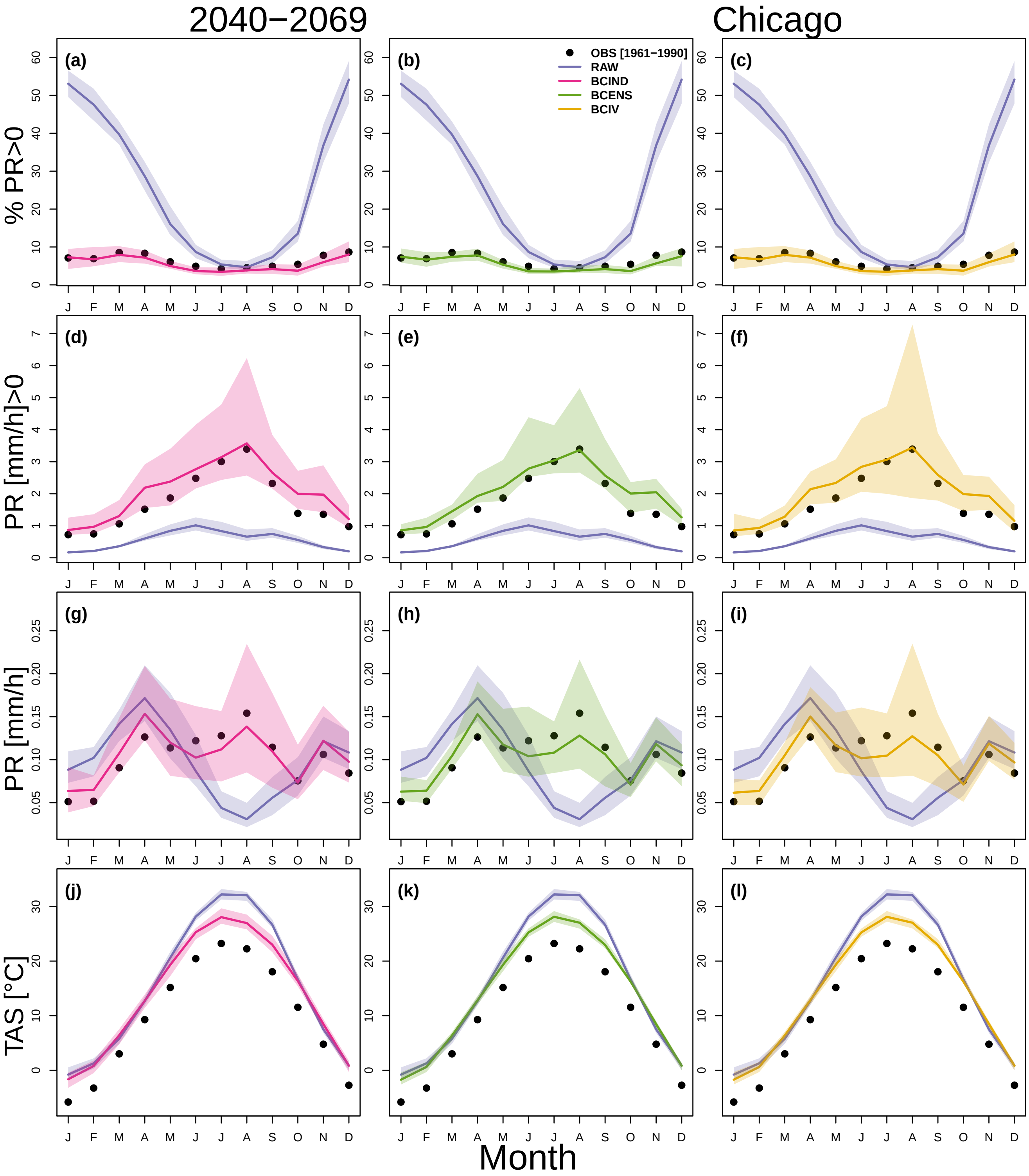

Figure S3. Idem Figure S1 for the 2040-2069 period.

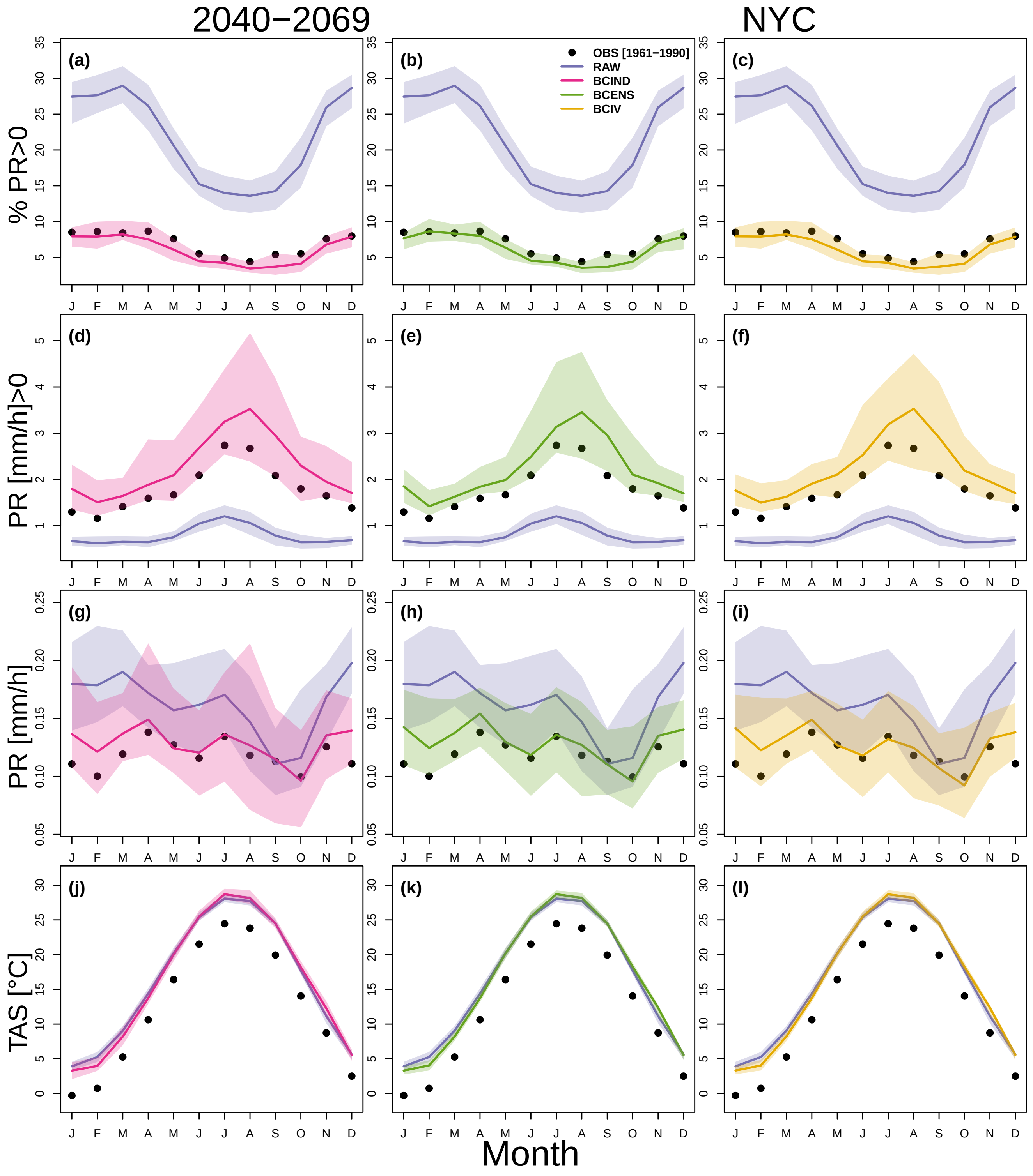

Figure S4. Idem Figure S2 for the 2040-2069 period.

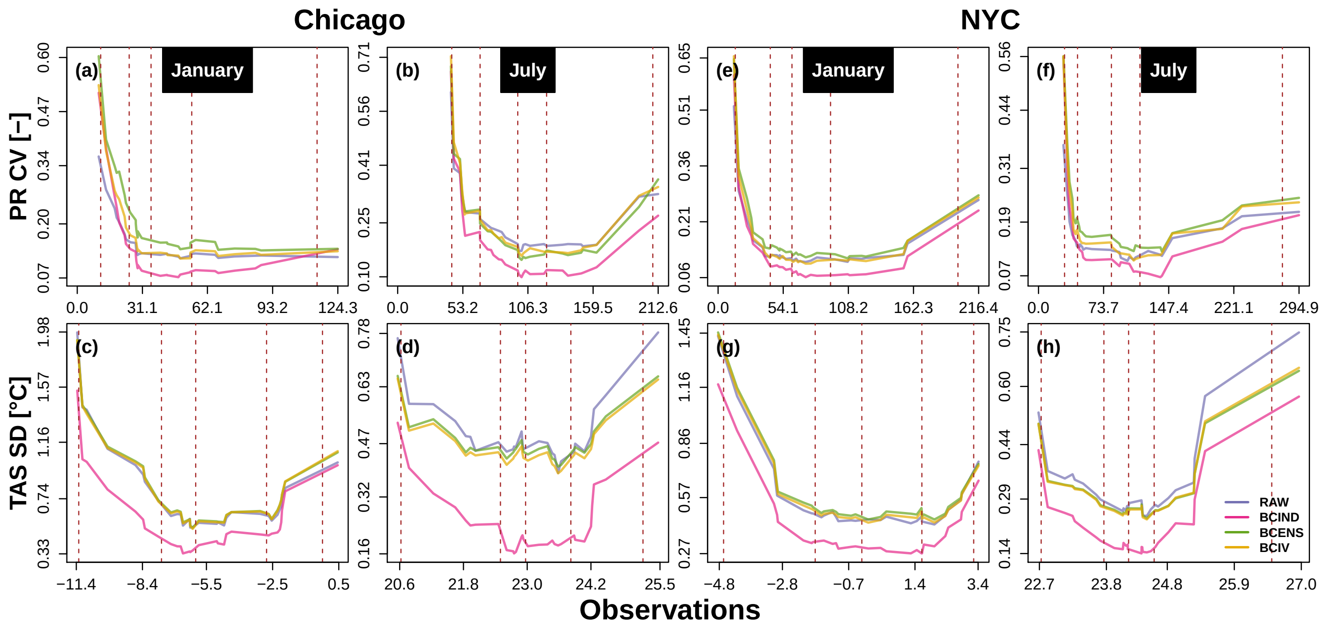

Figure S5. Same as Figure 4 of the article but for monthly total precipitation and mean temperatures. Vertical dashed lines represent the first, 25th, 50th, 75th and 99th percentiles for both variables.

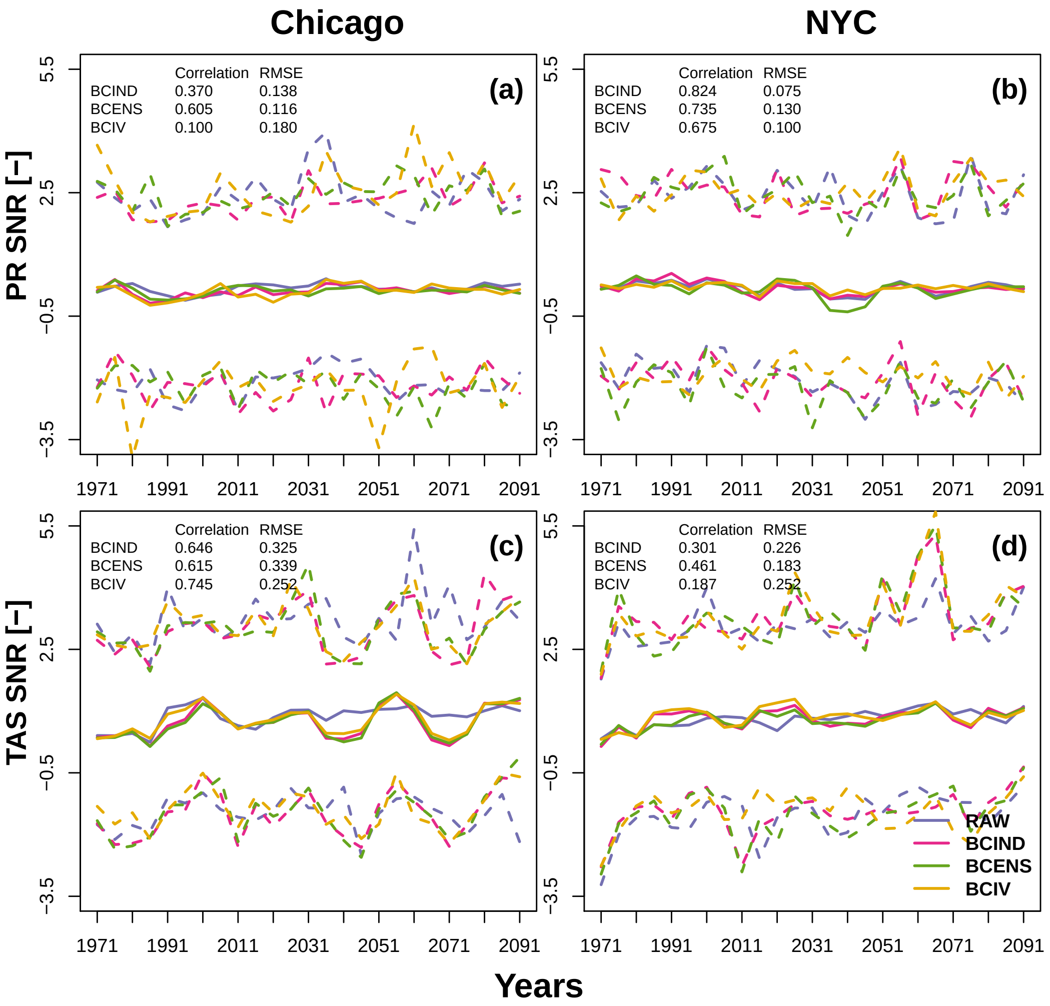

Figure S6. Same as Figure 5 of the article but for annual maximum precipitation and temperature.
